# Evaluation of a Computer-Aided Diagnosis System in the Classification of Lesions in Breast Strain Elastography Imaging

**DOI:** 10.3390/bioengineering5030062

**Published:** 2018-08-09

**Authors:** Karem D. Marcomini, Eduardo F. C. Fleury, Vilmar M. Oliveira, Antonio A. O. Carneiro, Homero Schiabel, Robert M. Nishikawa

**Affiliations:** 1 Department of Electrical and Computer Engineering, University of São Paulo, 400 Trabalhador São-carlense Av., São Carlos 13566-590, Brazil; homero@sc.usp.br; 2 Brazilian Institute for Cancer Control, 2576 Alcântara Machado Av., São Paulo 03101-005, Brazil; edufleury@hotmail.com; 3 Faculty of Medical Sciences of Santa Casa de São Paulo, 61 Doutor Cesário Motta Júnior St., São Paulo 01221-020, Brazil; vilmarmarques@uol.com.br; 4 Department of Physics, University of São Paulo, 3900 Bandeirantes Av., Ribeirão Preto 14040-901, Brazil; adilton@usp.br; 5 Department of Radiology, University of Pittsburgh, 3362 Fifth Av., Pittsburgh, PA 15123, USA; nishikawarm@upmc.edu

**Keywords:** breast cancer, elastography imaging, computer-aided diagnosis, color map, inter-observer agreement

## Abstract

**Purpose:** Evaluation of the performance of a computer-aided diagnosis (CAD) system based on the quantified color distribution in strain elastography imaging to evaluate the malignancy of breast tumors. **Methods:** The database consisted of 31 malignant and 52 benign lesions. A radiologist who was blinded to the diagnosis performed the visual analysis of the lesions. After six months with no eye contact on the breast images, the same radiologist and other two radiologists manually drew the contour of the lesions in B-mode ultrasound, which was masked in the elastography image. In order to measure the amount of hard tissue in a lesion, we developed a CAD system able to identify the amount of hard tissue, represented by red color, and quantify its predominance in a lesion, allowing classification as soft, intermediate, or hard. The data obtained with the CAD system were compared with the visual analysis. We calculated the sensitivity, specificity, and area under the curve (AUC) for the classification using the CAD system from the manual delineation of the contour by each radiologist. **Results:** The performance of the CAD system for the most experienced radiologist achieved sensitivity of 70.97%, specificity of 88.46%, and AUC of 0.853. The system presented better performance compared with his visual diagnosis, whose sensitivity, specificity, and AUC were 61.29%, 88.46%, and 0.829, respectively. The system obtained sensitivity, specificity, and AUC of 67.70%, 84.60%, and 0.783, respectively, for images segmented by Radiologist 2, and 51.60%, 92.30%, and 0.771, respectively, for those segmented by the Resident. The intra-class correlation coefficient was 0.748. The inter-observer agreement of the CAD system with the different contours was good in all comparisons. **Conclusions:** The proposed CAD system can improve the radiologist performance for classifying breast masses, with excellent inter-observer agreement. It could be a promising tool for clinical use.

## 1. Introduction

Breast cancer is the leading cause of cancer-related death in women [1]. Mammography and ultrasound (US) are commonly used for the detection and classification of breast masses in order to define the risk of malignancy. Both methods present some limitations. Mammography may yield false-negative results, especially in dense breasts, while US is sensitive in detection, but its specificity in lesion characterization is poor, leading to many unnecessary biopsy operations procedures and to radiologists failing to detect 10–30% of breast cancers [2,3,4,5].

Ultrasound elastography (UE) has been introduced as an additional modality for improving lesion classification [2]. This is an emerging technique that is considered equivalent of clinical manual palpation. Elasticity is one of the important characteristics of tissues that may change under the influence of pathologic processes, such as inflammation and tumor development [2,6]. Usually, a malignant lesion tends to be harder than a benign lesion because of its high cellularity and surrounding tissue desmoplasia [7,8]. 

**Figure 1:**
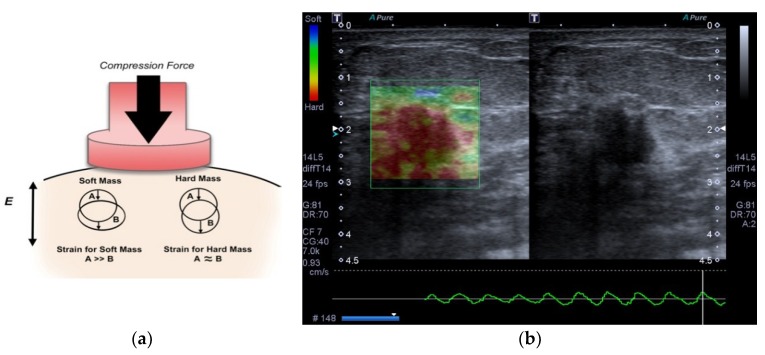
Strain elastography measures tissue displacement as a consequence of an applied initial compression. (**a**) Behavior of the soft and hard tissue after a compressive force. The displacement of the first is larger in soft tissue than hard tissue. (**b**) Image of invasive ductal carcinoma in a 56-year-old woman with strain elastography on left and B-mode ultrasound on right.

Strain elastography (SE) is a type of elastography based on applying a compressive force to the breast in order to assess the tendency of tissue to resist to deformation with an applied force, or to resume its original shape after removal of the force, thus providing a value of lesion stiffness in relation to the surrounding tissue [2,9,10,11]. Elastography is widely used to evaluate lesions detected at breast cancer screening [12,13]. The strain data are converted to images, often in the form of a color overlay upon the corresponding B-mode image, or a gray-scale image displayed next to the corresponding B-mode image. The side by side display without overlay allows a better appreciation of patterns of stiffness and softness within lesions as a result of the higher image contrast achievable when image transparency is not an issue [6,11]. In general, the elasticity information is displayed in the form of a gray image. The dark region of an elastogram indicates the hard tissue (no strain) and the bright one indicates the soft tissue (high strain). However, images can also be displayed in color scale, in which the color spectrum typically goes from blue tissue (high strain) to red (no strain), that is, from the soft to hard lesions, respectively, with an intermediate level green (with a medium level of strain). The color scale may vary depending on the ultrasound manufacturer [14]. Many studies have reported that it can increase the specificity of conventional B-mode ultrasound in differentiating benign from malignant masses, reducing the number of benign breast biopsy results [2,15]. An example of SE based compression process is shown in Figure 1.

The elasticity scores are generally assigned by the examiners as a qualitative classification and are not yet automated [16]. In the literature [17], the authors proposed a visual classification based on a three-point scale defined according to the color variation during compression and after decompression of the region of interest. The authors [17] determined a score of 1 for lesions that after decompression, the color spectrum identified as hard (i.e., red) covered less than 50% of the mass area when compared with the image acquired during compression. A score of 2 was assigned to the lesions that had hard tissue color variations covering between 50% and 90% after decompression. Finally, a score of 3 was assigned to the lesions with no significant color variation (hard tissue covering more than 90% of the mass area) during compression and decompression of the parenchyma. The same cutoff values were used in other elastography applications [18,19].

However, visual analysis results in significant inter-observer and intra-observer variability under the same conditions [16]. In the study proposed by the authors of [8], for example, three radiologists participated to classify 65 breast lesions (43 benign and 22 malignant). They used the Breast Imaging Reporting and Data System (BI-RADS) assessments for ultrasound, fat-to-lesion ratio, and elasticity score for elastography and they provided a combined diagnostic. Fair agreement (kappa = 0.37) was observed among them for BI-RADS assessment of ultrasound. Fair agreement with decreased kappa values (kappa = 0.25) was observed for combined ultrasound and elastography. Therefore, inter-observer variability is a key factor that can affect the diagnostic performance of elastography and B-mode ultrasound for breast tumor classification [20].

Some studies indicate the potential of quantitative evaluation of elastographic images in improving diagnostic accuracy, avoiding unnecessary biopsies, and proving to be useful in clinical diagnosis [15,16]. Computer-aided diagnosis (CAD) systems are emerging tools to give assistance in clinical use. In some studies, for example, the authors of [21,22,23,24,25,26] used the level set [27] for automatic segmentation on the lesion in the B-mode ultrasound. After that, the contour was mapped to the corresponding grey-scale elasticity image for the following calculation of tumor statistics. The color elastography was transferred to HSV (hue, saturation, value) color space and extracted meaningful features from hue images [23,28]. These features could be the following: (1) average tissue elasticity, sectional stiffness ratio, and normalized minimum distance [23]; (2) mean elasticity modulus, maximum elasticity modulus, standard deviation, hardness degree, and elasticity ratio [29]; (3) area difference, perimeter difference, contour difference, solidity, width to height difference, and texture features (standard deviation, energy, entropy, dissimilarity, homogeneity, and contrast) [30]. The quantified features were evaluated to determine if they were statistically significant in distinguishing between benign and malignant lesions. Most studies are aimed at the development of CAD systems for shear-wave eslatography [21,23,29] and only a few studies related to gray-scale SE [22,24,28]. To the best of our knowledge, a CAD system based on color spectrum SE has not yet been addressed.

Taking into account the visual classification described by the authors of [17,31] and the fact of having inter-observer variability in the elasticity scores as a visual (qualitative classification), we proposed to develop a new method to identify the hard area of a lesion in order to provide quantitative information, as well as classify the lesion according to the color predominance. We based the method on an innovative idea of creating a system that approximates the visual classification of the radiologist and reduces the diagnostic subjectivity of the radiologist. We validated our proposal by correlating the quantitative results provided by our system with the diagnosis assessed by a radiologists and histopathologic examination. We evaluated our method by the receiver operating characteristic (ROC) curve, sensitivity, specificity, and inter-observer agreement.

## 2. Materials and Methods

### 2.1. Image Database

The research ethics committee of the Brazilian Institute for Cancer Control (IBCC—São Paulo, SP, Brazil) approved this study (Protocol No. 012664/2016) and was registered in the Plataforma Brazil (Protocol No. 53543016.2.0000.0072). Investigators of the study obtained written informed consent from all included patients and protected their privacy. The collection of cases was from July to December 2015 during diagnostic breast exams at the IBCC. The data consisted of 83 consecutive women, represented by 92 solid lesions. All lesions underwent excisional biopsy; core needle biopsy; or fine-needle aspiration biopsy for pathologic diagnosis, used as the gold standard for evaluation of the CAD system. However, five patients were excluded because they presented non-mass lesions on the ultrasound before the percutaneous biopsy confirmation. A total of 83 lesions were included in the study, resulting in 31 malignant and 52 benign lesions.

The mean age of the patients submitted to the study was 46.5 years, ranging from 23 to 73 years (standard deviation of 8.6). The mean lesion size was 11 mm (ranging from 2.39 to 28.3 mm). Positive results for carcinoma were found in 6 patients younger than 40 years (19.3%), 11 patients between 40 and 50 years old (35.5%), and 14 patients older than 50 years (45.2%).

**Figure 2:**
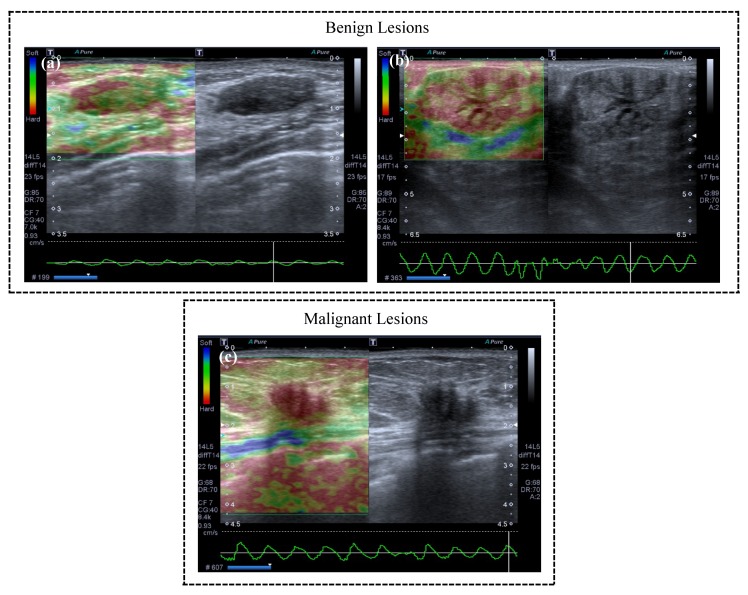
Examples of elastography imaging with different strain. The images were visually classified by a radiologist as follows: (**a**) soft; (**b**) intermediate; and (**c**) hard. The radiologist grouped lesions considered as (**a**) soft and (**b**) intermediate into negative cases (benign) and (**c**) hard lesions were classified as positive cases (malignant).

A radiologist with two years of experience performing breast ultrasound examinations obtained the conventional B-mode ultrasound and freehand strain elastography using a Toshiba Aplio 400 Ultrasound System (Toshiba, Tokyo, Japan) with a 5–10 MHz linear transducer. The scanning protocol in this research included transversal, longitudinal, radial, and antiradial real-time imaging of the target lesions with conventional US. The target mass was perpendicularly compressed with a tiny force applied by the transducer. The strain elastography image was superimposed onto the B-mode images with a color scale. The images that best represented the lesions using conventional ultrasound (lesion with the largest axis) and strain elastography (image with best quality standard for analysis according to the quality information of the equipment) were chosen. In the color scale (strain image), blue indicates soft tissue and red indicates hard tissue. B-mode images were on the right side and elastographic images were on the left side, as the examples illustrate in Figure 2. 

### 2.2. Delimitation of the Lesion

In elasticity imaging, automatic segmentation of lesions is a difficult task because of the distribution of colors, irregularity of the image, and difficulty in the direct extraction of the lesion contour [28]. Therefore, it was opted for the three radiologists to manually and arbitrarily delineate the contour of the lesions in the B-mode ultrasound images [28,29]. After manual delimitation of the mass on the B-mode image, the extracted tumor was masked to the corresponding elasticity image for the calculation of the hard area in the lesion.

### 2.3. Classification

**Figure 3:**
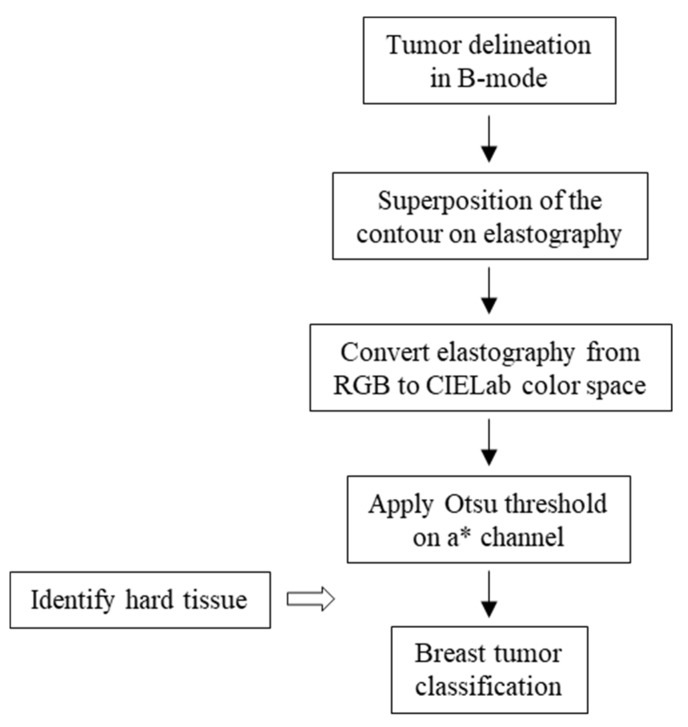
Computer-aided diagnosis system for breast tumor classification.

In order to measure the amount of hard tissue (i.e., tissues in red) in a lesion, we developed an algorithm for segmenting red areas and quantifying its predominance within the lesion, allowing us to classify it as soft, intermediate, or hard. The developed system is the result of an automatic process, in which the variable is the manual delimitation of the contour by the radiologist. The CAD system flowchart is presented in Figure 3. We compared the automatic classification with the percutaneous biopsy results. 

**Figure 4:**
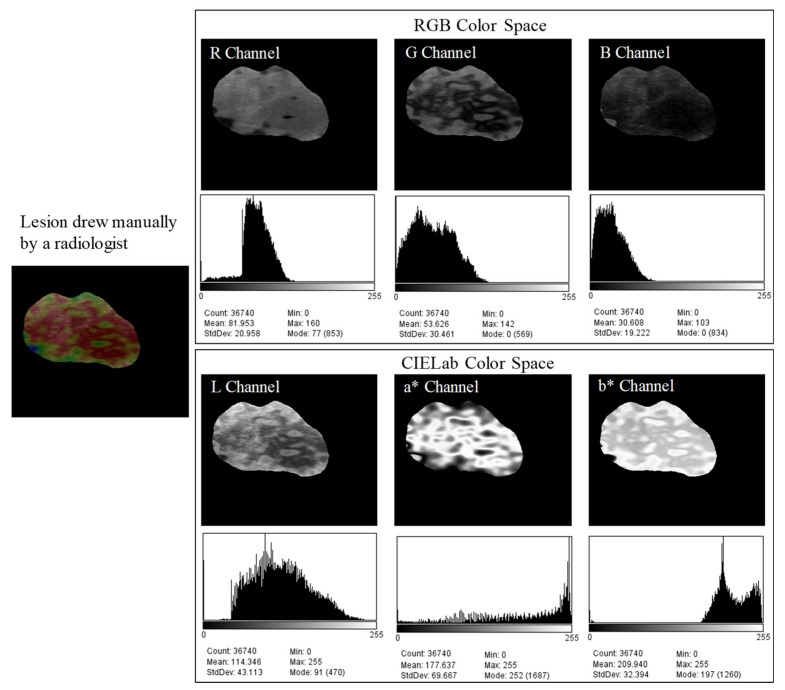
RGB and CIELab color space with their color channels shown separately.

A color model is a mathematical way of representing colors in numbers. There are several models and each one was derived for specific purposes and has certain advantages over the others. The main disadvantage of the RGB (red, green, blue) color space is related to the difficulty of recognizing different levels of intensity of the same chrominance. To avoid light intensity influence, we used the CIELab color space (also called L*a*b*) [32,33]. CIELab is a color space defined by the International Commission on Illumination (CIE) to express the color as three numerical values. It is represented by three matrices: brightness, green-red, and blue-yellow. The color axes are based on the fact that a color cannot be red and green or blue and yellow, because these colors oppose each other. On each axis, the values run from positive to negative (ranges from −127 to +127). On the a* axis, positive values indicate amounts of red, while negative values indicate amounts of green. On the b* axis, yellow is positive and blue is negative. The lightness or gray-scale axis (L*) represents the darkest black (L* = 0) and the brightest white (L* = 100). The asterisk (*) after L, a, and b are part of the full name to distinguish them from Hunter’s Lab color space [32,33]. Figure 4 illustrates an example of a fibroadenoma with the color channels from RGB and CIELab color space displayed separately. The contour was manually drawn by the radiologist and the adjacent tissues were removed from the image to improve the visualization of the mass. 

Analyzing the a* channel, we noted that it is possible to easily identify the red color, as it is represented by the lighter pixels. For this procedure, the Otsu method was applied [34] on the a* channel. The Otsu method is used to automatically perform clustering-based image thresholding or the reduction of a gray level image to a binary image. The algorithm assumes that the image contains two classes of pixels following bi-modal histogram (foreground pixels and background pixels), it then calculates the optimum threshold separating the two classes so that their combined spread (intra-class variance) is minimal, or equivalently (because the sum of pairwise squared distances is constant), so that their inter-class variance is maximal.

The cut-off point of classification is based on the percentage value of hard tissue in relation to the total area of the lesion. In order to find the best value of separation between benign and malignant lesions, we evaluated the performance of the CAD system using four values for the cut-off point. Table 1 shows the AUC obtained in each cut-off point.

Radiologist 1 performed a prior visual analysis of the lesions, and after six months with no eye contact with the images, he manually delimited the contour of the lesions. Data from the visual analysis are related to the clinical diagnosis provided by Radiologist 1, who did not use the computational system to assist in his diagnosis. However, Radiologist 1 was the only one who performed the visual analysis.

The best cut-off point achieved was 75% of hard lesions because it provided better AUC for two of the three classification tests, in which one of them was using the delimitation of the contour by the most experienced radiologist. 

After finding the cut-off point that would provide the best distinction between benign and malignant lesions, the lesion was classified as follows: (1) soft for lesions with red areas lower than 50% of the total area delineated by the observers; (2) intermediate for lesions with red areas between 50–75%; and (3) hard for red areas higher than 75% of the total lesion area. From the redefinition of classification values that had initially been proposed by the authors of [17], we achieved better diagnostic accuracy for this threshold values, as shown in previous work [35]. We considered lesions classified as soft and intermediate to be benign and hard to be malignant.

**Figure 5:**
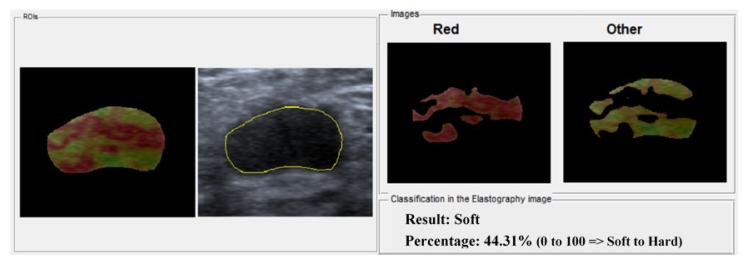
Classification using the system proposed.

As output, the system provides two images: an image with the region classified as hard (red region) and another image with the region classified as soft (other colors). Thus, the radiologist can analyze the reliability of the computational diagnosis. Furthermore, the system also provides the percentage value of hard tissue in the lesion, representing the tendency of malignancy, in which 0% corresponds to benign lesions and 100% to malignant lesions, as shown in Figure 5. 

### 2.4. Data Evaluation and Statistical Analysis

Two radiologists, with 16 and 10 years of experience, and a second-year resident in imaging diagnosis, drew the contour of the lesions and assisted in evaluating our algorithm. 

We compared the manual delimitation of the observers using the following measures: the Jaccard Similarity Index (JSI) [36], oversegmentation (AVM) [37], and undersegmentation (AUM) [37]. 

JSI was used to compare the similarity of a data set, and was defined by the ratio between the intersection and the union of two areas (Equation (1)).

(1) $$\mathrm{JSI}=\frac{A_{seg}\cap A_{gt}}{A_{seg}\cup A_{gt}}$$

where $A_{seg}$ denotes the segmented area and $A_{gt}$ is the corresponding ground truth area. JSI ranges from 0 to 1, and the higher the value, the better the segmentation result. 

Jaccard does not provide information on the undersegmentation and oversegmentation. So, AUM and AVM were measured and are defined by Equations (2) and (3).

(2) $$\mathrm{AUM}=\;\frac{A_{gt}-A_{seg}}{A_{gt}}$$

(3) $$\mathrm{AVM}=\;\frac{A_{seg}-A_{gt}}{A_{seg}}$$

Values of AUM and AVM range from 0 to 1, and large values indicate undersegmentation or oversegmentation, respectively.

The kappa coefficient [38] was used to measure the inter-rater agreement, in which 0.0–0.2 was considered poor, 0.21–0.40 fair, 0.41–0.60 moderate, 0.61–0.80 good, and 0.81–1.00 very good agreement. We calculated the sensitivity, specificity, and ROC curves for all the observers. The ROC curves were obtained using bootstrapping with 95% confidence intervals (CI) in all cases and were compared using a significance level of 5%. For the calculation of the kappa coefficient, sensitivity, specificity, and AUC, we used the Med Calc software v16.2 (MedCalc Software, Ostend, Belgium), with significant levels at *p* < 0.05.

## 3. Results

### 3.1. Manual Delineation

**Figure 6:**
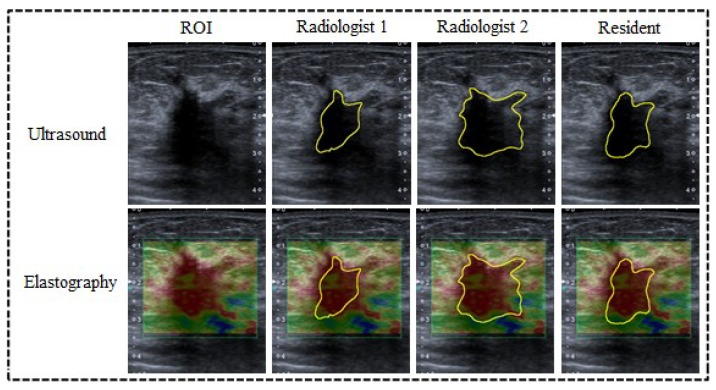
Results of the manual delineation of the tumor on B-mode image and contour mapped on the elasticity image.

Figure 6 illustrates the manual delimitation of the lesion on the B-mode ultrasound and the superposition of the contour in the elastography image. 

The radiologists were blinded to the diagnosis when they manually delimited the contour of the lesion. Table 2 gives the agreement between the three radiologists’ manual delineation, using different measures. 

The desired value corresponds to the perfect overlap (JSI = 1.0) between the considered segmented areas, with neither oversegmentation (AVM = 0) nor undersegmentation (AUM = 0).

The highest conformity in manual delineation occurred between Radiologist 1 and the Resident, wherein they obtained the highest overlap index (JSI of 0.654) and low indices of undersegmentation and oversegmentation (0.227 and 0.169, respectively). On the other hand, the lowest conformity was between Radiologist 2 and the Resident, considering mainly the high index of undersegmentation (0.402) of the Resident in relation with Radiologist 2. Values are expressed in more detail in Table 2.

### 3.2. Classification Evaluation

We used the sensitivity, specificity, and the area under the ROC curve (AUC) measure to evaluate the performance of the CAD system with the contour delineated by each specialist. Table 3 shows the results of the classification using the cut-off of 75%. 

Table 4 shows the final classification of the lesions using the CAD system from the contour delineated of each radiologist according to the histological diagnosis (benign or malignant). 

The mean and median values correspond to the classification score. Score 1 was assigned to soft lesions, score 2 to intermediate lesions, and score 3 to hard lesions.

### 3.3. Statistics Analysis

Table 5 shows the inter-observer agreement (kappa coefficient) for all the observers. The intra-class correlation coefficient was 0.748.

We used the kappa coefficient to evaluate the concordance in the diagnosis of the CAD system with the contour of the lesion performed by specialists with different levels of experience in order to verify the susceptibility of our system to the contour. Based on the results presented in the table above, the agreement was good in all cases. The best agreement was found in the comparison between the CAD system with the contours of Radiologists 1 and 2.

Table 6 compares the differences in AUC values between all the radiologists, considering the visual analysis provided by Radiologist 1 and the automatic classification based on the manual delineation of each one. 

The table above shows that there is practically no inter-observer variation in the AUC. Although slight, the greatest variation is noted in the comparison between Radiologist 1 and Resident. This is because of the difference in experience time between them.

## 4. Discussion

The American College of Radiology suggests elasticity assessment as a way to evaluate breast tumor malignancy in the fifth edition of Breast Imaging Reporting and Data System (BI-RADS), released in 2013 [39]. The suggestion indicates that elastography provides additional diagnostic information over conventional B-mode imaging. This is in order to improve differentiation of benign and malignant breast tumors and avoid unnecessary biopsy.

The analysis of strain images involves the evaluation of the distribution of colors within the lesion and allows the classification of these lesions as soft, intermediate, or hard, as proposed by the BI-RADS lexicon. The inter-observer agreement is uncertain in the strain method because it is operator dependent and the results are qualitative.

This study presented a CAD system based on SE elastography images that could enable objective evaluation of the hard tissues of breast masses. The CAD system proposed is an innovative method that attempts to classify the lesion in a similar way to the specialist’s visual classification and provides quantitative information regarding the hard tissues present within the lesion. 

In a CAD system, the accurate segmentation of breast lesions in US images is a difficult task, mainly in automatic systems, as a result of presence of speckle noise and shadows, the low or non-uniform contrast of certain structures, and the variability of the echogenicity of the masses, while manual delineation is a subjective, time-consuming, and operator-dependent procedure.

We performed an initial study to evaluate the manual segmentation of the radiologists, because errors or distortions in the lesion representation may result in misdiagnosis. In our study, Resident (less experienced observer) presented manual delineation closer to Radiologist 1 (the most experienced), with a higher Jaccard index (0.654) and lower oversegmentation (0.169). When we measured inter-observer agreement, the observers obtained a good value (kappa = 0.758). On the other hand, Radiologist 2 did not produce manual delineation as close to Radiologist 1 as that of Resident, with a similar inter-observer agreement (kappa = 0.796). We consider that the change in contour was not a harmful factor in the classification.

The computer method was compared with the physician’s visual diagnosis. The system provided an increase in sensitivity (70.97% vs. 61.29%) and the specificity remained constant (88.46%), compared with the visual assessment by Radiologist 1 (see Table 3). The AUC value was 0.853 for the case using the new method by computer, and 0.829 for the visual assessment. In relation to Radiologist 2 and Resident, they reached AUCs of 0.806 and 0.814, respectively. Furthermore, we achieved good agreement among all observers, indicating that the CAD system can aid in the interpretation of the elastography image. 

This research is an apparent improvement of a previous work [35], in which we developed another color classification approach. The previous system was more user-dependent and presented significant inter- and intra-observer variation. This is because the user had to manually delimit a part of the red region, which may affect classifying the lesion. Based on the exposed, the algorithm had already presented results [35] comparable to the visual classification of the radiologist with sensitivity, specificity, and an AUC value of 54.5%, 90.5%, and 0.837, respectively.

In the study proposed by Chang [28], they evaluated the performance of a new computer-aided method on color strain elastography in the differentiation of benign and malignant breast lesions. Their method consisted of the conversion from RGB to HSV color space and the extraction of six features (tumor mean, inner mean, outer mean, hard rate, inner hard, and outer hard) from hue images. Then, the neural network was utilized to distinguish tumors. The proposed CAD system presented better performance than the physician with 85.07% of sensitivity and 83.19% of specificity in comparison with 53.73% of sensitivity and 92.92% specificity from the physician. The kappa statistics was applied to measure the agreement between the proposed CAD system and the physician’s diagnosis, the kappa of 0.54 indicated the moderate agreement between observers. Lo [22] presented an approach of CAD system for gray-scale strain elastography. They extracted the contour using pre-processing techniques for contrast enhancement and level set to detect the edge of the lesion. The fuzzy c-means method was used to classify the pixels into three clusters and define the stiff area. Six strain features were extracted and the leave-one-out cross-validation method was used to validate the performance of the selected subset. As a result, the diagnostic performance of the CAD system achieved values of 80%, 80%, and 0.84 for sensitivity, specificity, and AUC, respectively.

One factor to consider is the lack of a public breast elastography image database. As the performance of the algorithms is dependent on the images that are collected for each work, results cannot be reliably compared.

The limitations of this study include the fact that the entire dataset was used in the training process and the lack of an automatic segmentation method. Another limitation is that the proposed system is based on the detection and quantification of the red color, whose prevalence determines the degree of stiffness of a lesion. A possible improvement of the CAD system could be the inclusion of the B-mode features, such as those related to the morphology and texture of the lesion, in addition to the use of machine learning techniques. On the other hand, the system is simple, fast, and achieves similar or better results among the mentioned works, proving to be an important tool for classifying breast lesions in elastography images. 

## 5. Conclusions

The proposed CAD system can reduce the inter-observer variability for breast elastography. The CAD system had a similar performance to the diagnosis of the most experienced radiologist, which would provide promising diagnoses in clinical use. In future work, we intend to evaluate the visual analysis of more radiologists, in order to include automatic segmentation techniques, as well as to include more features and study other types of automatic classifiers, such as machine learning techniques. In addition, an ultrasound image characterization system could be included in our CAD system in order to provide more information about the lesion, increasing the diagnostic accuracy in classifying breast elastography images.

## Figures and Tables

**Table 1:** Classification with different cut-off point. AUC—area under the curve.

Observers	AUC—70% of Hard Tissue	AUC—75% of Hard Tissue	AUC—80% of Hard Tissue	AUC—90% of Hard Tissue
**Radiologist 1**	0.841	0.853	0.802	0.790
**Radiologist 2**	0.813	0.806	0.815	0.707
**Resident**	0.802	0.814	0.789	0.723
**Visual Analysis—Radiologist 1**	0.829			

**Table 2:** Measures to evaluate the manual delineation.

Observers		Jaccard	Undersegmentation	Oversegmentation
Radiologist 1 and Radiologist 2	Average	0.565	0.147	0.355
	SD	0.178	0.144	0.213
Radiologist 1 and Resident	Average	0.654	0.227	0.169
	SD	0.122	0.148	0.135
Radiologist 2 and Resident	Average	0.537	0.402	0.144
	SD	0.193	0.212	0.174
Mean Value Desired		1.000	0.000	0.000

**Table 3:** Classification of the lesions based on the manual delimitation of radiologists.

Observers	Sensitivity	Specificity	AUC
**Radiologist 1**	70.97	88.46	0.853
**Radiologist 2**	67.74	84.62	0.806
**Resident**	58.06	90.38	0.814
**Visual Analysis—Radiologist 1**	61.29	88.46	0.829

**Table 4:** Distribution of the final classification according to the score adopted, where score 1 represents soft lesions; score 2 represents intermediate; and score 3 is associated with hard lesions.

	Type	*n*	Radiologist 1		Radiologist 2		Resident	
			Median	Mean	Median	Mean	Median	Mean
Benign (*n* = 48)	Fibrocystic changes	30	2.0	1.7	1.0	1.7	1.5	1.7
	Fibroadenoma	18	2.0	1.6	2.0	1.6	2.0	1.6
Malignant (*n* = 31)	Invasive ductal carcinoma	23	3.0	2.5	3.0	2.5	3.0	2.4
	Invasive lobular carcinoma	2	3.0	3.0	3.0	3.0	3.0	3.0
	Ductal Carcinoma in Situ (DCIS)	4	3.0	3.0	2.5	2.5	2.5	2.5
	Others	2	2.5	2.5	2.5	2.5	2.5	2.5
Indeterminate (*n* = 4)	Indeterminate lesions	4	2.0	1.8	2.0	2.3	2.0	2.0
**Total**		83	-					

**Table 5:** Inter-observer agreement in the diagnosis of lesions in elastography imaging using the computer-aided diagnosis (CAD) system with different contours.

Observers	Kappa
Radiologist 1 and Radiologist 2	0.796
Radiologist 1 and Resident	0.758
Radiologist 2 and Resident	0.682

**Table 6:** Classification based on automatic system.

Observers	Difference in AUC	95% Confidence Intervals (CI)	*p*-Value
Visual Analysis—Radiologist 1	0.024	−0.048–0.096	0.517
Visual Analysis—Radiologist 2	0.023	−0.050–0.096	0.538
Visual Analysis—Resident	0.033	−0.047–0.113	0.420
Radiologist 1 and Radiologist 2	0.047	−0.024–0.118	0.196
Radiologist 1 and Resident	0.057	−0.015–0.128	0.120
Radiologist 2 and Resident	0.010	−0.081–0.101	0.830
